# A random forest classifier for detecting rare variants in NGS data from viral populations

**DOI:** 10.1016/j.csbj.2017.07.001

**Published:** 2017-07-19

**Authors:** Raunaq Malhotra, Manjari Jha, Mary Poss, Raj Acharya

**Affiliations:** a The School of Electrical Engineering and Computer Science, The Pennsylvania State University, University Park, PA, 16802, USA; b Department of Biology, The Pennsylvania State University, University Park, PA 16802, USA; c School of Informatics and Computing, Indiana University, Bloomington, IN 47405, USA

**Keywords:** Sequencing error detection, Reference free methods, Next-generation sequencing, Viral populations, Multi-resolution frames, Random forest classifier

## Abstract

We propose a random forest classifier for detecting rare variants from sequencing errors in Next Generation Sequencing (NGS) data from viral populations. The method utilizes counts of varying length of *k*-mers from the reads of a viral population to train a Random forest classifier, called MultiRes, that classifies *k*-mers as erroneous or rare variants. Our algorithm is rooted in concepts from signal processing and uses a frame-based representation of *k*-mers. Frames are sets of non-orthogonal basis functions that were traditionally used in signal processing for noise removal. We define discrete spatial signals for genomes and sequenced reads, and show that *k*-mers of a given size constitute a frame.

We evaluate MultiRes on simulated and real viral population datasets, which consist of many low frequency variants, and compare it to the error detection methods used in correction tools known in the literature. MultiRes has 4 to 500 times less false positives *k*-mer predictions compared to other methods, essential for accurate estimation of viral population diversity and their *de-novo* assembly. It has high recall of the true *k*-mers, comparable to other error correction methods. MultiRes also has greater than 95% recall for detecting single nucleotide polymorphisms (SNPs) and fewer false positive SNPs, while detecting higher number of rare variants compared to other variant calling methods for viral populations. The software is available freely from the GitHub link https://github.com/raunaq-m/MultiRes.

## 1 Introduction

The sequence diversity present in a population of closely related genomes is important for their survival under environmental pressures. Viral population within a host is an example of such population of closely related genomes, where some viral strains survive even when large segments of their genome are deleted. The sequence variants that occur at low frequency in the population, also known as rare variants, have been known to impact the population's survival and understanding their prevalence is important for drug design and in therapeutics [1].

However, detection of rare variants from Next Generation Sequencing (NGS) data is still a challenge as the rare variants are tangled with errors in sequencing technologies due to their similar prevalence [2], [3]. The NGS data technologies are error prone and even though their error profiles are well studied [4], [5], removing sequencing errors is essential before downstream processing of NGS data such as assembly of haplotypes in viral populations [2], [6], [7], [8], [9] and variant calling for viral populations [7], [9], [10].

In order to remove sequencing errors from NGS data, the first step is detecting the errors from true biological sequences and then correcting the errors to the true sequence. For NGS data obtained from a viral population, the reads are mapped to a reference genome to detect true variants from sequencing errors based on a probabilistic model [6], [7], [9], [10], [11], and then the sequencing errors are corrected to the sequence of the reference genome. However, as virus population contains a large diversity of true sequences, accurate mapping of reads to any one reference may not be possible.

Alternatively, sampled reads are broken into small fixed length sub-strings called *k*-mers and their counts are used for error detection (e.g. [12], [13], [14], [15], [16]). The erroneous *k*-mers are corrected by changing minimum number of bases in the reads using the detected true *k*-mers. These methods use a generative model for *k*-mer counts to determine if an observed *k*-mer is erroneous or a true *k*-mer [12] based on a counts threshold [12], [13], [14], [17].

For *k*-mer based error detection, the length of the *k*-mer and the frequency threshold are important parameters. The size of a *k*-mer can effect the performance of error detection method, as it either decreases the evidence for a segment of the genome for a large *k*, or combines evidences from multiple segments for a small *k*. However, a single appropriate *k*-mer size for error detection in viral populations is restrictive in nature, as a combination of different sized overlapping *k*-mers, although redundant, can provide richer information.

The error detection part in most *k*-mer based error correction tools [12], [13], [14], [15] has been designed assuming the reads are sampled from a single diploid genome and rely on a single counts threshold. However, for viral populations a single threshold is not suitable as viral strains occur at different relative frequencies. Currently, a number of time and memory efficient *k*-mer counting algorithms are available [18], [19]. Thus, choosing an appropriate size of *k*-mer is possible by performing *k*-mer counts at multiple sizes [20].

With the availability of large amounts of data from NGS technologies, data driven classifiers have also been used for detection of sequencing errors [21] and for variant calling [5], [12], [22], [23]. However, identifying the features for classification of rare variants and sequencing errors is still a challenge, due to their similar characteristics in the NGS data.

We propose, MultiRes, a reference-free *k*-mer based error detection algorithm for a viral population. The algorithm uses *k*-mer counts of different sizes to train a Random Forest Classifier that classifies *k*-mers as erroneous or rare variant *k*-mers. We also propose a mechanism for selecting the optimal combination of *k*-mer sizes. The rare variant *k*-mers along with high frequency *k*-mers can be used as is in downstream tools for variant calling and for de novo assembly of viral populations.

MultiRes uses a collection of sizes of *k*-mers as features for detecting sequencing errors and rare variants. Our rationale to choose a combination of sizes for *k*-mers is rooted in signals processing, where analysis of signals at different resolutions has been used for noise removal [24]. Signals are projected onto a series of non-orthonormal basis functions, known as a **frame**, [25], [26], [27]; these projections are used for error removal and signal recovery [28], [29].

The classifier in MultiRes is trained on a simulated dataset that models NGS data generated from a replicating viral population. We evaluate the performance of MultiRes on simulated and real datasets, and compare it to the error detection algorithms of error correction tools BLESS [13], Quake [12], BFC [14], and Musket [15]. We also compare our results to BayesHammer [30] and Seecer [31], which can handle variable sequencing coverage across the genome and polymorphisms in the RNA sequencing data respectively.

MultiRes has a high recall of the true *k*-mers, comparable to other methods and has 5 to 500 times better removal of erroneous *k*-mers compared to other methods. Our results demonstrate that the classifier in MultiRes performs well for error detection on real sequencing data obtained from the same sequencing technology. Thus, the classifier in MultiRes is generalizable to viral population data from the same sequencing technology.

As MultiRes detects the rare variant *k*-mers in an NGS data, its output can be directly used for identifying rare variants in a viral population. Variant calling for viral populations typically relies on a single reference genome or on a consensus genome generated from the population being studied [7], [9], [10]. We compare the rare variants detected by MultiRes to variant calling methods VPhaser-2 [9], LoFreq [10] and the outputs from haplotype reconstruction method ShoRAH [7]. MultiRes has the higher recall of true SNPs compared to the SNPs called by VPhaser-2, LoFreq and ShoRAH on both simulated and real datasets, and misses the least number of true SNPs amongst all methods. This demonstrates its applicability for rare variant detection in viral populations.

## 2 Methods

MultiRes is a classifier for detecting sequencing errors from rare variants. The counts of the *k*-mers along with the counts of their sub-sequences (sub *k*-mers within a *k*-mer) are used as features for training a classifier. The true *k*-mers observed in the viral haplotypes with counts in the reads less than a threshold *T*_*High*_ are defined to be rare variant *k*-mers, while the rest of *k*-mers with counts less than *T*_*High*_ are erroneous *k*-mers. The *k*-mers that occur at counts greater than *T*_*High*_ are known as common *k*-mers, as they occur frequently in the viral haplotypes. The common *k*-mers are assumed to be error-free and the classifier is trained only for the erroneous and rare variant *k*-mers.

The premise of our method is that reads sequenced from a population of genomes can be modeled as discrete spatial signals. Discrete spatial signals can be projected on to a **frame** [25], [26], [27], [32] for their representation (See Supplementary Material for details), where the coefficients of projections characterize the discrete spatial signals. Similarly, we show that *k*-mers (of a given size *k*) form a **frame** and the maximal projection of *k*-mers correspond to their counts in a sequencing run. Additionally, a *k*-mer can be projected on to a collection of **frames**, where each **frame** represents counts of *k′*-mers (*k′* < *k*) that are sub-strings of the given *k*-mer.

The choice of *k* for a **frame** is important and should be large enough such that a *k*-mer only occurs once in the haplotypes. On the other hand, it should be smaller than the read lengths so that *k*-mer counting is still meaningful.

The minimum *k* can be approximated by ensuring that the probability of picking a string of length equal to the genome length (say |*H*|) where all *k*-mers in it occur only once is low [12]. Thus the probability of picking approximately |*H*| unique *k*-mers out of a set of 4^*k*/2^ (considering reverse complements) should be low. We set 2 ⋅|*H*|/4^*k*^ ≈ *ϵ*, where *ϵ* is a small number, to determine the smallest possible choice of *k* (*k*_*min*_) for the **frame**.

As an example, a *k*-mer *u* occurring *c*(*u*) times in the reads when projected on a **frame** of size *k* is in-fact represented by its maximal projection *c*(*u*). The same *k*-mer *u*, can also be represented on **frames** of sizes (*k′*,*k*^*′′*^ in the range [*k*_*min*_,*k*]. Now the maximal projections for *u* in these **frames** are the counts of *k′*-mers and *k*^*′′*^-mers present within *u*. This representation of *k*-mer *u* can be used to train a classifier for identifying erroneous versus rare variant *k*-mers.

### 2.1 MultiRes: Classification algorithm for detecting sequencing errors and rare variants

We define a classifier, *EC*, for classifying a *k*-mer as erroneous, a rare variant, or a common *k*-mer in the dataset. Algorithm 1 describes MultiRes, the proposed algorithm for detecting rare variants and sequencing errors. The algorithm takes as input the sampled reads, the classifier *EC*, an ordered array (*k*,*k′*,*k*^*′′*^), and a threshold parameter *T*_*High*_. It outputs for every *k*-mer observed in the sampled reads a status: whether the *k*-mer is erroneous or a rare variant.

It first computes the counts of *k*-mers, *k′*-mers, and *k*^*′′*^-mers using the dsk *k*-mer counting software [18]. The *k*-mers *u* that have counts greater than *T*_*High*_ are marked as true *k*-mers while the rest of the *k*-mers are classified using the classifier *EC* based on their counts on *k′*-mers, and *k*^*′′*^-mers.

The classifier *EC* captures the profile of erroneous versus rare variant *k*-mers from Illumina sequencing of viral populations. We used the software dsk (version 1.6066) [18] for *k*-mer counting, which can perform the *k*-mer counts in a limited memory and disk space machine quickly. The run time of MultiRes is linearly dependent on the number of unique *k*-mers in a dataset, as once the classifier *EC* is trained, it can be used for all datasets, and it can be easily parallelized.

Algorithm 1 MultiRes: Error detection in the sampled reads by **frame**-based classification of *k*-mers

**Figure:**
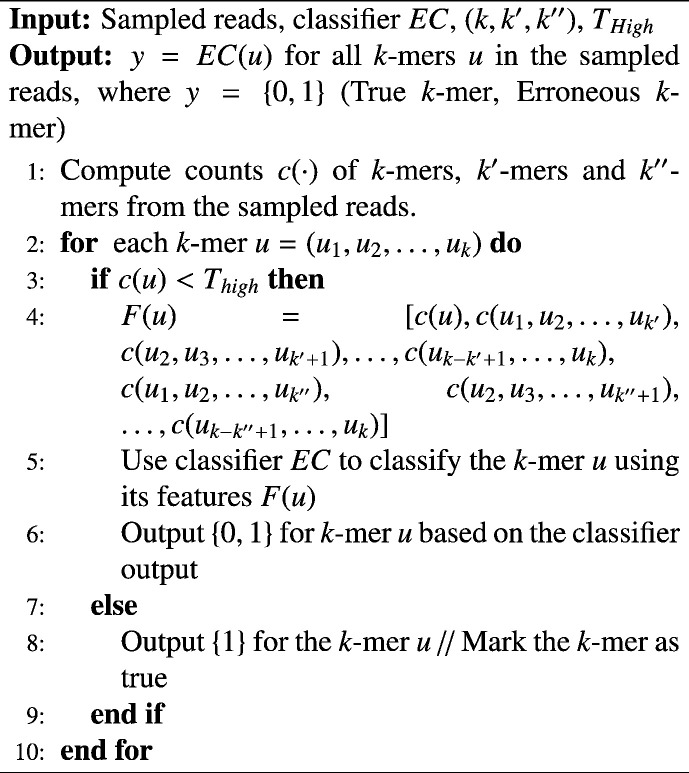

#### 2.1.1 Simulated data for classifier training

MultiRes assumes the availability of a classifier *EC* which can distinguish between the erroneous and rare variant *k*-mers. We use simulated datasets to train a series of classifiers and set *EC* to the classifier which has the highest accuracy. The simulated viral population consists of 11 haplotypes and is generated by mutating 10% of positions on a known HIV-1 reference sequence of length 9.18 kb (NC_001802). These mutations also model the evolution of a viral population under a high mutation rate. The mutations introduced are randomly and uniformly distributed across the length of the genome so that the classifier is not biased towards the distribution of true variants. This introduces a total of 195,000 ground truth unique 35-mers in the simulated HIV-1 dataset.

We next simulate Illumina paired-end sequencing reads using the software dwgsim (https://github.com/nh13/DWGSIM) at 400x sequencing coverage from this viral population. The status of each *k*-mer in this dataset is known as being erroneous, rare variant or a common *k*-mer. We use close to 100,000 *k*-mers in the training dataset. Thus, there is a test dataset of *k*-mers left for evaluating the efficacy of the classifiers.

In order to train a classifier, we need to choose the size of the *k*-mer, the sizes of *k′*-mers for computing the projections of *k*-mer signals, and the number of such projections needed. The choice of the smallest of {*k*,*k′*,…} should be above the minimum length *k*_*min*_ to ensure that each *k*-mer still corresponds to a unique location on a viral genome.

For HIV populations, with genome length 9180 base pairs (9.1 kbp) and taking *ϵ* = 0.001 (a small value, as mentioned before), the minimum length of *k*-mer is *k*_*min*_ = ⌈log _4_2 ⋅ *G*/*ϵ*⌉ = ⌈12.06⌉ = 13. As in signal processing domain, we choose *k* ≈ 3 ⋅ *k*_*min*_ = 35 (an integral multiple of *k*_*min*_) as the largest *k*-mer, and consider its projections on **frames** of sizes ranging from 13 to 35.

MultiRes assumes that *k*-mers above the threshold count *T*_*high*_ are error-free, and only classifies the *k*-mers with counts less than *T*_*high*_. The choice of *T*_*high*_ should ensure that the probability of erroneous *k*-mers with counts above *T*_*high*_ is negligible. We use the gamma distribution model mentioned in the Quake error correction paper [12] for modeling erroneous *k*-mers, as it approximates the observed distribution of errors. Based on this gamma distribution, we set *T*_*high*_ = 30 for the simulated HIV population data. The classifiers are therefore trained on 35-mers with counts less than 30.

**Fig. 1:**
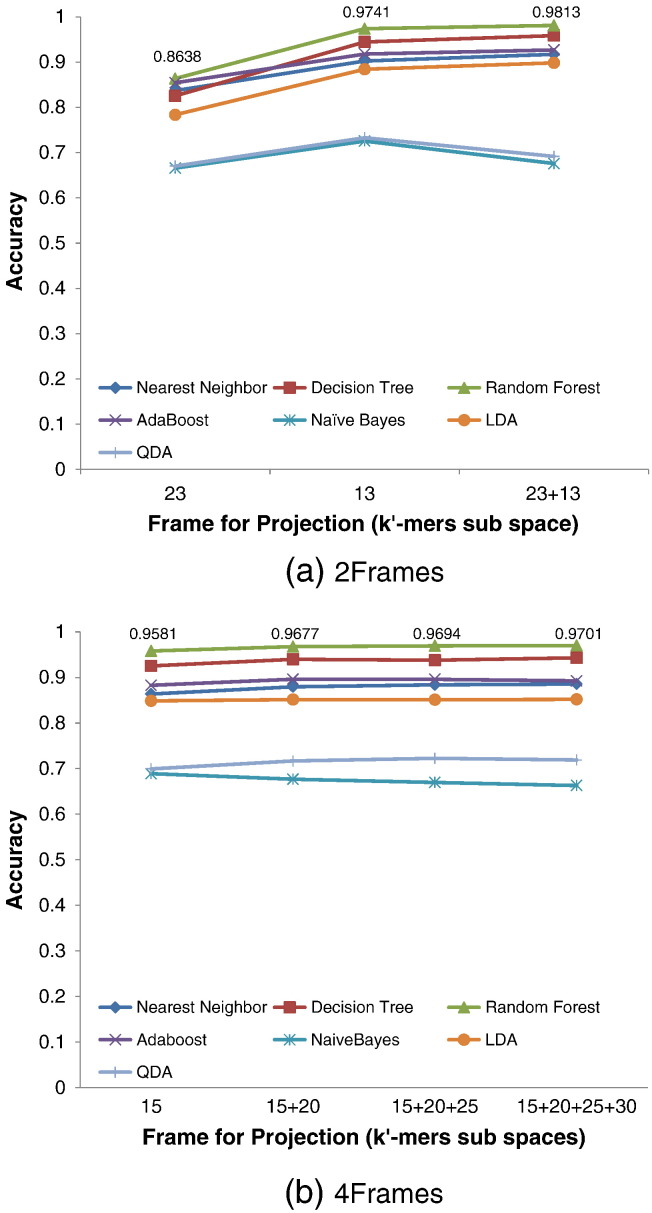
Performance of classification algorithms for erroneous versus rare variant *k*-mer classification. The performance of mentioned classification algorithms for classifying 35-mers are compared over two sets of features. 35-mers are either projected onto a family of (a) 23-mers, 13-mers, and a 13 + 23-mers, and (b) projections onto 15-mers, 15 + 20-mers, 15 + 20 + 25-mers, and 15 + 20 + 25 + 30-mers. The accuracy reported is over fivefold cross validation on 35-mers extracted from HIV viral population. Accuracy improves when 35-mers are projected onto smaller sized *k′*- mers and as the number of projections increases. Random Forest Classifier has the best accuracy across different classification algorithms.

Three training datasets consisting of both erroneous and rare variant 35-mers are generated. The features in the three datasets are the projection of the 35-mers onto (i) the frame of size 23, (ii) the frame of size 13, and (iii) a combination of both frames (Fig. 1 a). The features in the three settings translate to the counts of the 13-mers and 23-mers observed within the 35-mer along with the counts of the 35-mer. We observed 11.9 million unique 35-mers in the simulated HIV-1 population, from which features from 76,000 erroneous 35-mers and 32,000 true variant 35-mers distributed uniformly over counts 1–30 were used for training the classifiers.

#### 2.1.2 Classifier selection

Classifiers Nearest Neighbor, Decision Tree, Random Forest, Adaboost, Naive Bayes, Linear Discriminant Analysis (LDA), and Quadratic Discriminant Analysis (QDA) are trained on the three training datasets and evaluated based on their test data accuracy over a 5-fold cross validation dataset. The classifiers are implemented in the scikit-learn library (version is 0.16.1) in python programming language (version 2.7.6). For all the classifiers, the accuracy improves as the 35-mers are projected onto 13-mers rather than 23-mers (higher resolution, lower size of *k′*-mers), and improves even further when 35-mers are resolved onto both 13-mers and 23-mers (Fig. 1). No further feature selection was performed when 35-mers were resolved onto 13-mers and 23-mers. The Random Forest Classifier performs the best on all three datasets, where the accuracy for dataset (iii) is 98.12*%*. The accuracy for Naive Bayes and QDA classifiers are lower for all datasets, and also decreases when the projections in both 13-mers and 23-mers are considered, indicating that inadequacy of their models for the classification of 35-mers in these projections. The performance of other classifiers are comparable and follows similar trends.

#### 2.1.3 Exploring additional feature spaces

Additionally, we generate a series of 4 projections of the 35-mers onto frames of sizes a) 15, b) {15 + 20}, c) {15 + 20 + 25}, and d) {15 + 20 + 25 + 30} to evaluate the effect of number of frames used for projection on the performance (Fig. 1 b). Increasing the number of projections has no visible effect on increasing the accuracy of performance, although it increases the memory requirements and time complexity for computing counts of all five different values of *k*. Based on this, we chose the Random Forest classifier with a resolution of 35-mers decomposed into a combination of 13-mers and 23-mers for other simulated and real datasets.

## 3 Results

### 3.1 Error detection for reconstruction of haplotypes

We evaluate MultiRes on simulated HIV and HCV datasets and a laboratory mixture of HIV-1 strains. MultiRes is compared to the detection algorithms in the error correction tools Quake (last checked version Feb 2012) [12], BLESS (version 0.15) [13], Musket (last downloaded October 2015) [15], BFC (last downloaded October 2015) [14], BayesHammer (version 3.6.2) [30] and Seecer (version 0.1.3) [31]. As these tools are traditionally designed for error correction, the error corrected reads or *k*-mers from these methods were used for comparison with the rare variant *k*-mers and common *k*-mers predicted by MultiRes. ShoRAH [7] reconstructs a set of haplotypes as its final output rather than error corrected reads and thus was not evaluated for error correction. The error corrected reads, although available as an intermediate output, are not reported due to their low precision numbers, but ShoRAH is used for single nucleotide variant calling and comparison later in the text. Other recent error correction methods available for viral populations such as PredictHaplo http://bmda.cs.unibas.ch/software.html, HaploClique [11], and Viral Genome Assembler (VGA) [8] were not evaluated in this study.

Three measures, defined in terms of the true and erroneous *k*-mers, are used for comparing the detection algorithms in all methods. *Precision* is defined as the ratio of the known true *k*-mers identified to the total number of *k*-mers predicted as true variants by an algorithm. *Recall* is defined as the ratio of the true variant *k*-mers identified to the total number of true *k*-mers by an algorithm and measures the goodness of a method to retain true *k*-mers for a dataset. *False Positives to True Positives Ratio* (FP/TP ratio) is the ratio of the erroneous *k*-mers predicted as true variants to the true variant *k*-mers identified by the algorithm. FP/TP ratio measures the number of erroneous *k*-mers identified by an algorithm to detect a single true variant *k*-mer and is a measure of the overall volume of *k*-mers predicted by an algorithm.

#### 3.1.1 HIV simulated datasets

We first assess the performance of MultiRes on the reads simulated from the HIV-1 population containing 11 haplotypes, generated from a single HIV-1 reference sequence (NC_001802) as mentioned before. Two datasets are generated from the simulated reads: one with average haplotype coverage of 100x (denoted as HIV 100x), and second where the average coverage is 400x (denoted as HIV 400x) as increasing sequencing depth increases the absolute number of erroneous *k*-mers introduced in the data.

The recall of MultiRes is 95% and 98% on HIV 100x and HIV 400x datasets, respectively, indicating that performance of MultiRes improves with increasing sequencing depth as expected. The recall numbers are comparable to around 98% recall of other methods on the HIV 100x dataset and 94% to 99% for HIV 400x dataset (Table 1).

**Table 1:** Comparison of performance metrics of error detection on simulated HIV datasets. FP/TP ratio is the measure of false positive to true positive ratio, Recall measures the percentage of true *k*-mers out of all true *k*-mer predicted by an algorithm, Precision measures the percentage of predicted *k*-mers by an algorithm that are true *k*-mers.

Algorithm	FP/TP ratio		Recall		Precision	
	HIV 100x	HIV 400x	HIV 100x	HIV 400x	HIV 100x	HIV 400x
Uncorrected	53	121	98.91	99.67	1.85	0.82
Quake	9.26	29.5	**98.63**	94.84	9.74	3.27
BLESS	0.71	76.7	98.38	99.36	58.48	1.28
Musket	0.46	121	98.46	**99.67**	68.48	0.82
BFC	2.12	112	98.47	99.57	32.01	0.89
BayesHammer	0.37	69.1	98.47	98.59	73.04	1.42
Seecer	12.1	110	98.49	98.31	7.65	0.90
MultiRes	**0.11**	**0.048**	95.01	98.17	**89.34**	**95.39**

The False positive/True Positive ratios (FP/TP ratios), Recall, and Precision are compared on two HIV datasets for the methods: Quake, BLESS, Musket, BFC, BayesHammer, Seecer, and the proposed method MultiRes. The error corrected reads from each method are broken into *k*-mers and compared to the true *k*-mers in the HIV-1 viral populations. Uncorrected denotes the statistics when no error correction is performed. Bold in each column indicates the best method for the dataset and the metric evaluated.

The precision of MultiRes is 89% in the HIV 100x while all other methods have low precisions for HIV 100x. While precision in all other methods is less than 5% for HIV 400x dataset, the precision of MultiRes is 95%, suggesting that precision decreases for other methods with increasing sequencing depth. As higher depth samples also have higher sequencing errors, the detection algorithms in these methods are not able to differentiate between rare variants and sequencing errors. Seecer and BayesHammer, methods which can handle variability in sequencing coverage, also have very low precision values compared to the proposed method.

The FP/TP ratio obtained by MultiRes are 4 to 500 times better than other methods and the number of *k*-mers retained is close to the true set of *k*-mers in the two datasets (FP/TP ratio is close to zero & recall close to 95–98 %).Thus, while all methods retain the true *k*-mers to the same extent, only MultiRes reduces the number of false positive *k*-mers. This is important as the memory requirements for *de novo* assembly tools linearly increases based on the number of *k*-mers. Thus the *k*-mers predicted by MultiRes would have a500 times reduction in memory consumption for downstream *de novo* assembly tools as compared to current error correction methods.

#### 3.1.2 Generalizability: Testing MultiRes on a Hepatitis C virus dataset

We also evaluate our method on reads simulated from viral populations consisting of the E1/E2 gene of Hepatitis C virus (HCV). The purpose of using HCV strains is to understand the generalization of the MultiRes classifier on other viral population datasets. Two HCV populations observed in patients in previous studies are used as simulated viral populations. The first, denoted as HCV 1, consists of 36 HCV strains from E1/E2 region and are of length 1672 bps [33]. The second, denoted as HCV 2, consists of 44 HCV strains from the E1/E2 regions of the HCV genome with lengths 1734 bps [8], [17]. We simulate 500 K Illumina paired end reads from both datasets under a power law (with ratio 2) of reads distribution amongst the strains [34]. The two simulated datasets are denoted as HCV1P and HCV2P respectively. The power law distribution of reads also helps in evaluating the performance of MultiRes when more than 50% of the haplotypes are present at less than 5% relative abundances.

All methods have recall greater than 90% on both datasets (Table 2). Again, the difference between MultiRes and other methods is evident from the FP/TP ratios and precision. The false positive to true positive ratios for MultiRes are less than other methods at least by a factor of 5 (Table 2). MultiRes still outperforms all other methods on predicting the smallest set of predicted *k*-mers while maintaining high recall levels of true *k*-mers.

**Table 2:** Comparison of performance metrics of different methods on HCV population datasets.

Algorithm	FP/TP ratio		Recall		Precision	
	HCV1P	HCV2P	HCV1P	HCV2P	HCV1P	HCV2P
Uncorrected	1201	571	99.51	99.88	0.08	0.17
Quake	303.3	149	96.41	97.23	0.32	0.66
BLESS	202	112	98.35	97.18	0.49	0.88
Musket	938	463	93.53	89.17	0.10	0.21
BFC	352	161	99.32	99.84	0.28	0.61
BayesHammer	699	340	98.12	97.1	0.14	0.29
Seecer	1095	528	**99.48**	**99.85**	0.09	0.19
MultiRes	**37.4**	**19.54**	96.5	94.25	**2.6**	**4.87**

The false positive to true positive ratios, Recall, and Precision of error correction methods on the two simulated HCV datasets are shown. Uncorrected refers to the statistics when no error correction is performed. Bold font in each column indicates the best method for each dataset on the evaluated measure.

The recall for MultiRes is respectively 96% and 94% on HCV1P and HCV2P datasets, which is less than the method Seecer that has recall values around 99%. Seecer marks more than 90% of the observed *k*-mers as true, which explains the high recall values. However, this also leads to a large number of false positive *k*-mers being predicted as true *k*-mers in Seecer, leading to low precision values. All other methods also achieve high recall by retention of all large fraction of observed *k*-mers, as indicated by their precision values being less than 1% and false positive to true positive ratios being greater than 100.

The similar performance of MultiRes on a dataset, such as the HCV population, which is diverse in genome composition from the simulated HIV-1 sequences used in simulation indicates the generalizability of the Random Forest Classifier in MultiRes. The classifier is capturing properties of the Illumina sequencing platform and the fact that both datasets contain a large number of rare variants occurring at *k*-mer counts close to the sequencing errors. Thus, MultiRes can be used as it is for error and rare variant detection in diverse datasets.

**Fig. 2:**
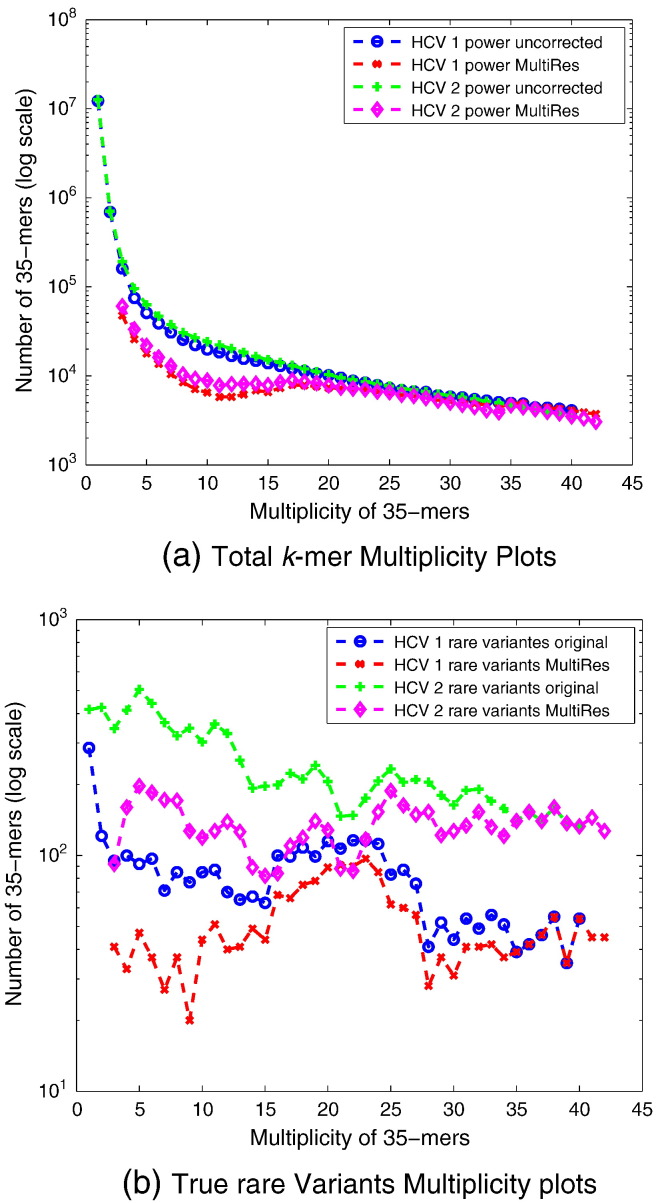
Performance of MultiRes on HCV datasets under power law distributions of viral haplotypes with respect to count of *k*-mer. 35-mer multiplicity plots for HCV1P and HCV2P datasets are shown. x-axis indicates the number of times a 35-mer was observed while y-axis indicates the number of 35-mers at a count. (a) The predicted true 35-mers from MultiRes (HCV1P red, HCV2P pink) compared to the uncorrected data (HCV1P blue,HCV2P green), and (b) The true positive rare variants 35-mers from MultiRes (HCV1P red, HCV2P pink) versus the ground truth 35-mers (HCV1P red, HCV2P pink). MultiRes predicts rare variants *k*-mers at all counts greater than 3, with its accuracy improving as counts of *k*-mer increases.

As the performance of MultiRes on HCV population is not as impressive as on the HIV simulated populations, it is also important to understand the cause for this decrease in performance. It is possible that the decrease in performance is correlated to the large number of low-frequency variants that are being misclassified by MultiRes. In order to test this, we investigate MultiRes' classification as a function of the count of the 35-mer which is being classified. MultiRes predicts about one-fourth of the observed *k*-mers as rare variants for *k*-mer counts less than 15, and predicts more than 99% all of the observed *k*-mers as true for counts greater 20 (Fig. 2 (a)). This suggests that MultiRes predicts rare variant *k*-mers for all observed counts and detects more rare variant *k*-mers than a method based on a single threshold.

Most of the *k*-mer based error correction methods use a single threshold over the *k*-mer counts, which will clearly lose true rare variant *k*-mers (Fig. 2 (b)). On the other hand, MultiRes has a recall of 50% for *k*-mers observed 3 times, while still correctly identifying more than 75% of the *k*-mers as erroneous. The recall of MultiRes increases to 100% as the counts of the observed *k*-mers increases to 35. This indicates the importance of not having a single threshold for distinguishing between sequencing errors and rare variants in viral population datasets, and our MultiRes bypasses a single threshold by training a Random Forest classifier.

#### 3.1.3 Evaluation on population of 5 HIV-1 sequences

We also evaluate MultiRes on a laboratory mixture of five known HIV-1 strains [35], which captures the variability occurring during sample preparation, errors introduced in a real sequencing project, and mutations occurring during reverse transcription of RNA samples. Five HIV-1 strains (named YU2, HXB2, NL43, 89.6, and JRCSF) of lengths 9.1 kb were pooled and sequenced using Illumina paired end sequencing technology (Refer to [35] for details). Each HIV strain was also sequenced separately in their study and aligned to their known reference sequence (from Genbank) to generate a consensus sequence for each HIV-1 strain [35]. This provides us with a dataset of actual sequence reads where the ground truth is known allowing us to assess the performance of MultiRes and other methods. We extracted 35-mers from the paired end sequencing data and classify them using the Random Forest classifier of MultiRes trained on the simulated HIV sequencing data.

All the error correction methods and MultiRes have recall values around 97%, indicating that the performance for recovery of true *k*-mers is comparable across all methods (Table 3). The false positive to true positive ratio for MultiRes is 13 while all other methods have ratios more than 120. MultiRes predicts 359 thousand unique *k*-mers in the set of true *k*-mers while all other methods predict more than 5 million unique *k*-mers. Even methods that take variance in sequencing depths while performing error correction, such as BayesHammer and Seecer, predict 11.3 million and 6.3 million unique *k*-mers which is two orders more than the ground truth number of *k*-mers in the consensus sequence of the 5 HIV-1 strains (53 thousand unique *k*-mers). Thus, even considering the artifacts introduced in sequencing, MultiRes has by far the most compact set of predicted error free *k*-mers amongst all methods while retaining high number of true *k*-mers. As mentioned earlier, as the number of *k*-mers linearly affects the memory requirements for downstream *de novo* assembly methods, the error detection from MultiRes would translate to a 10-fold reduction in memory.

#### 3.1.4 Runtime and memory

**Fig. 3:**
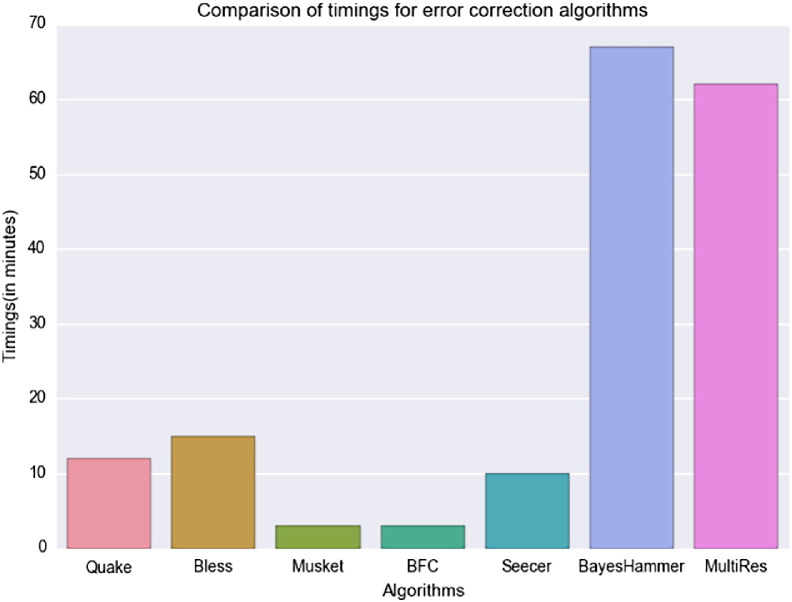
Runtime comparison on five-viral mix dataset. Comparison of running times for different algorithms on 5-viral mix dataset on 8GB memory nodes of 2X Dual Core AMD Opteron 2216 systems from Dell. The time noted for BayesHammer is only the time reported for BayesHammer error correction step in SPADES (version 3.6.2). The time reported for MultiRes is the combined time for *k*-mer counting, predicting *k*-mers as erroneous and rare variants and generating the final output.

MultiRes has comparable running times to BayesHammer on the five-viral mix dataset (Fig. 3) on a Dell system with 8 GB main memory, and 2X Dual Core AMD Opteron 2216 CPU type. The performance on all other datasets was similar indicating that the timings are comparable. Additionally, while other methods have parallel implementations, the error detection classifier step in MultiRes is a single thread serial implementation. As the random forest classifier used by MultiRes is already trained and independent of the input *k*-mers for classification, the runtime of MultiRes can be significantly improved via parallelization of the *k*-mer classification step.

### 3.2 Comparison of MultiRes to variant calling methods for viral populations

As one of the objectives in NGS studies of viruses is to identify the single nucleotide polymorphisms (SNPs) in a population [2], [7], [9] which is sensitive to erroneous reads, we evaluate the inference of SNPs from the *k*-mers predicted by MultiRes, and compare it to known SNP profiling methods for viral populations. We first align the predicted *k*-mers from MultiRes to a reference sequence of the viral population using the bwa mem aligner and a base is called as a SNP when its relative fraction amongst the *k*-mers aligned at that position is greater than 0.01. All the variants that occur at a frequency greater than the error threshold at that position are reported as SNPs. The choice of the reference sequence is based on the viral population data being evaluated, and the same reference sequence is used for calling true SNPs and the SNPs predicted by a method.

Each SNP detected at a base position of the reference and detection of the reference base itself are treated as *true positives* for a method; thus the number of true positives can be greater than the length of the reference sequence. All the SNPs predicted by a method and the number of bases mapped to the reference sequence are known as the *total SNP predictions* of a method. We use three measures for evaluating the SNPs called by any method. *Precision* is defined as the ratio of the number of *true positives* to the *total SNP predictions* made by a method, while *recall* is defined as the ratio of the *true positives* to the total number of SNPs and reference bases in the viral population. Finally, *false positive to true positive ratio* is a ratio of the number of false SNP predictions to the number of *true positives* detected by a method.

We compare our results to state-of-the-art variant calling methods for viral populations VPhaser-2 [9], a rare variant calling method LoFreq [10], and viral haplotype reconstruction algorithm ShoRAH [7] using the above three measures. The reference sequence used by variant calling methods VPhaser-2 and LoFreq is the same as that used by *samtools* to determine the true SNPs, while the SNPs predicted by ShoRAH at default parameters are compared directly to the true SNPs. We only used the SNP calls from VPhaser-2 for evaluation, as length polymorphisms are not generated by the other methods, but the results from VPhaser-2 were not penalized when comparing the SNPs.

We report results for LoFreq [10], VPhaser [9], ShoRAH [7] and our method MultiRes on all datasets (Table 4). Overall, MultiRes has greater than 94% recall and precision values greater than 83% in all datasets. LoFreq and VPhaser have comparable recall but lower precision values and an increase in the FP/TP ratios on the HCV population datasets, indicating a decrease in performance. ShoRAH overall has lower recall values, nevertheless a 100% precision in all but the 5-viral mix dataset, suggesting that it misses true SNPs but is very accurate when it calls a base as SNP. Overall all methods have low values for FP/TP ratio as compared to before, indicating that the number of false positive SNP predictions are low. The metric where MultiRes outperforms others is the lowest number of true SNPs missed. This shows that even with a simplistic SNP prediction method used in MultiRes, it is able to capture the true variation of the sampled viral population and has the lowest false negatives of well established methods. This demonstrates that using error-free set of *k*-mers can vastly increase the variant detection in viral populations.

**Table 3:** Comparison of performance metrics on 5-viral mix HIV-1 dataset.

Algorithm	Recall	Precision	FP/TP ratio	# of unique 35-mers
Uncorrected	98.01	0.2	439	11.4 M
BLESS	97.31	0.4	227	5.89 M
Musket	**97.91**	0.3	366	11.2 M
BFC	97.55	0.3	316	9.6 M
BayesHammer	97.49	0.8	122	6.3M
Seecer	97.84	0.5	220	11.3M
MultiRes	96.64	7.1	**13**	**359 K**

The recall, precision, and FP/TP ratios of each method are evaluated on the 5-viral mix HIV-1 dataset. The number of unique 35-mers indicates the number of unique 35-mers predicted by a method. There are 53 thousand true unique 35-mers in the consensus sequences of the 5 viral strains. Bold indicates the best method for the measure in each column.

**Table 4:** Comparison with Variant Calling methods on all datasets.

Dataset	Method	Recall (%)	FP/TP ratio	Precision (%)	# of False negatives	Mapped reads (%)
HIV 100x	LoFreq	97.33	0.004	99.60	444	89.51
	Vphaser	98.90	0.007	99.26	183	89.51
	ShoRAH	55.21	**0**	**100**	7746	**98.04**
	MultiRes	**99.69**	0.011	98.88	**51**	97.89
HIV 400x	LoFreq	84.83	0	99.99	2522	99.55
	Vphaser	**95.92**	0.292	77.37	**678**	99.55
	ShoRAH	55.21	**0**	**100**	7746	**99.95**
	MultiRes	95.57	0.007	99.33	736	97.34
HCV1P	LoFreq	**98.30**	1.282	43.82	**31**	**99.99**
	Vphaser	93.51	1.628	38.05	118	**99.99**
	ShoRAH	91.92	**0**	**100**	147	**99.99**
	MultiRes	98.24	0.597	62.64	32	97.32
HCV2P	LoFreq	97.10	1.046	48.87	60	**100**
	Vphaser	95.65	1.492	40.13	90	**100**
	ShoRAH	83.73	**0**	**100**	337	99.95
	MultiRes	**98.79**	0.201	83.27	**25**	85.14
5-viral mix	LoFreq	99.06	0.085	92.15	101	98.59
	Vphaser	92.68	0.039	96.25	789	98.59
	ShoRAH	98.66	**0.014**	**98.99**	109	**99.3**
	MultiRes	**99.39**	0.077	92.82	**66**	96.29

The Recall, false positive to true positive ratios (FP/TP), Precision, number of false negatives, and % of mapped reads by methods LoFreq, VPhaser-2, ShoRAH, and MultiRes are computed for listed datasets. All reads from a sample were aligned using bwa-mem tool for LoFreq and VPhaser-2 under default settings. ShoRAH uses its own aligner for read alignment and variant calling, while *k*-mers detected by MultiRes were aligned using bwa-mem for MultiRes. Outputs from LoFreq (version 2.1.2), VPhaser-2 (last downloaded version October 2015), and ShoRAH (last downloaded version from November 2013) are compared against known variants for simulated datasets. For 5-viral mix, the consensus reference provided by [35] was used to determine ground truth variants. MultiRes variants are determined by aligning 35-mers to a reference sequence and bases occurring at more than 0.01 frequency as variants. Bold for each dataset indicates the best method for the performance measures.

The number of reads or *k*-mers aligned to the reference sequence are comparable across the methods, except for HCV2P dataset where MultiRes has 85% *k*-mers mapped compared to 100% read mapping (Table 4). It is possible that the unmapped *k*-mers correspond to the length variants and could be verified by haplotype reconstruction using the predicted *k*-mers, but that was not the focus in this paper.

## 4 Discussion and conclusions

We have proposed a classifier MultiRes for detecting rare variant and erroneous *k*-mers obtained from Illumina sequencing of viral populations. Our method does not rely on a reference sequence and uses concepts from signal processing to justify using the counts of sets of *k*-mers of different sizes. We utilize the projections of sampled reads signals onto multiple **frames** as features for our classifier MultiRes.

We demonstrated the performance of MultiRes on simulated HIV and HCV viral populations and real HIV viral populations containing viral haplotypes at varying relative frequencies, where it outperformed the error detection algorithms used in error correction methods in terms of recall and the total number of predicted *k*-mers. Though, the error detection algorithms in the error correction methods evaluated assumed that sequenced reads originated from a single genome sequenced at uniform coverage, our method also works better than the method BayesHammer, which can tackle non-uniform sequencing coverage, and the method Seecer, which additionally incorporates methods for detecting alternative splicing and polymorphisms.

The error-free *k*-mers predicted by MultiRes enable the usage of *de novo* assembly methods for viral genomes. A major challenge for using De Bruijn graph based methods for viral populations has been the increased complexity of the graph due to the presence of large number of sequencing errors [36]. Moreover, the memory footprint of a De Bruijn assembly graph increases linearly with the number of *k*-mers in the NGS data. Thus the low false positives along with high recall of *k*-mers predicted by MultiRes drastically reduce the memory requirements for De Bruijn graphs. An edge-centric De Bruijn graph of size *k* − 1 can be directly generated from error free *k*-mers, such as in *de novo* assembly tools SPADES, Cortex [37], [38] for reconstruction of viral haplotypes in a viral population. The graph can be used for calling structural variants in the viral population data. MultiRes has high recall of true *k*-mers while outputting the least number of false positive *k*-mers, thereby making *de novo* assembly graphs manageable.

MultiRes also can be directly used for SNP calling as the predicted error-free *k*-mers can be aligned to an existing reference genome or a consensus sequence of the current viral population. The SNPs called by MultiRes' data has either the highest or the second highest recall of the SNPs compared to other methods for viral population variant calling.

MultiRes relies on the counts of multiple sizes of *k*-mers observed in the sequenced reads, and the choice of *k*-mer length is an important parameter. The minimum value of *k* chosen should be such that a *k*-mer can only be sampled from a single location in the genome. This is possible in viral populations where there are small repeats present. Choosing the number of *k*-mer sizes used is another parameter, and while accuracy can be improved by increasing it, additional *k*-mer counting increased the number of computations. As demonstrated by our experiments, choosing three different values of *k*, namely (*k*,2 ⋅ *k*,3 ⋅ *k*) was sufficient for accurate results.

MultiRes also has applications for studying the large scale variation in closely related genomes, including as viral populations. The complexity of De Bruijn graphs, useful for studying structural variants and rearrangements in the population, increases because of sequencing errors. Our method can provide a compact set of *k*-mers while still retaining high recall of the true *k*-mers, which can be utilized for constructing the graph. Additionally, the error-free *k*-mers predicted by MultiRes can be directly used for understanding the SNPs observed in the viral population to a high degree of accuracy.

MultiRes' classifier also has its limitations. The model, although trained to model the features of an Illumina sequencing machine, does have a decreased performance on different viral populations with a large number of rare variants, as is evident from its 50% accuracy for HCV2P population for *k*-mers observed only 3 times. Although it is able to eliminate a large number of false positive *k*-mers (more than 75% of *k*-mers at counts of 3), the classifier model can be improved with additional training data and an ensemble of classifier models.

MultiRes was primarily developed for detection of sequencing errors and rare variants in viral populations, which have small genomes. Extending our method for larger genomes may require additional tuning of the parameters via re-training of the classifier, but the concepts developed here are applicable to studying variation in closely related genomes such as cancer cell lines. It is also applicable for understanding somatic variation in sequences as their variation frequency is close to the sequencing error rates. The technique can also be explored for newer sequencing machines, such as PacBio sequences and Oxford Nanopore long read sequencing, where the type of sequencing errors are different, but the concepts of projections of signals are still applicable. The software is available for download from the github link (https://github.com/raunaq-m/MultiRes).

## Conflict of interest

The authors declare no conflict of interest.
