# Supplementary material for: A random forest classifier for detecting rare variants in NGS data from viral populations
Source: Comput Struct Biotechnol J. 2017 Jul 19;15:388–95. doi: 10.1016/j.csbj.2017.07.001 (PMC5548337; doi:10.1016/j.csbj.2017.07.001)
Supplement: Supplementary file 1 — Additional Methods. [file mmc1.pdf]

Supplementary Note: A classification based algorithm for removing errors to detect rare variants in NGS data

1. Supplementary Material

1.1. Definitions

1.1.1. Definition of Frames

A family of functions $\{f_m\}_{m\in\mathbb{N}}$ in a Hilbert Space $\mathcal{H}$ is a **frame** for $\mathcal{H}$ if there exists positive constants α and β such that

$$\alpha \cdot \|f\|^2 \leq \sum_m |\langle f, f_m\rangle|^2 \leq \beta \cdot \|f\|^2 \tag{1}$$

for all $f \in \mathcal{H}$ (Duffin and Schaeffer (1952); Kaiser (2010); Daubechies et al. (2003, 1986)). Here, $|\langle f, f_m\rangle|$ is the inner product of the function f with a function f_m in the family, while $\|f\|^2$ is the inner product of the function f with itself in the space $\mathcal{H}$, also known as the norm of f. The functions in $\{f_m\}_{m\in\mathbb{N}}$ are not necessarily orthogonal to each other. Thus, $|\langle f, f_m\rangle|$ or the projections form a redundant representation of the function f on the family $\{f_m\}_{m\in\mathbb{N}}$. Redundant projections of a signal have been exploited for noise reduction in signal processing Ferreira (1999).

1.1.2. Genomes as Spatial Signals

The genomic sequence of a haplotype in a viral population can be represented as a discrete spatial signal with a non-zero signal at a spatial position representing the base observed at the corresponding genomic position. Notationally, a haplotype H of length $|H|$ is represented as a discrete 4-dimensional spatial signal $\{H_n\}$ where the signal at the nth position is defined by a 4-dimensional vector $H[n] = (x_A[n], x_C[n], x_G[n], x_T[n])'$ for $n = \{0, 1, 2, \ldots, |H| - 1\}$. The element $x_b[n]$ corresponds to the base $b \in \{A, C, G, T\}$ observed at position n of the genome. A haplotype's spatial signal is represented by $\{H_n\}$, or H in short, and $H[n]$ is used to denote its nth sample in the rest of the paper.

For the spatial signal $\{H_n\}$ of a single haplotype two conditions hold true at each position n: (i) $x_b[n] \in \{0,1\}$, and (ii) $\sum_b x_b[n] = 1$ for $b \in \{A, C, G, T\}$. In other words, the non-zero entry in the 4-dimensional vector $(x_A[n], x_C[n], x_G[n], x_T[n])'$ at position n indicates that the corresponding base is observed at that position.

For a viral population $\mathbf{H}$ containing a collection of viral haplotypes, $\mathbf{H} = \{H_1, H_2, \ldots, H_P\}$, we can define a spatial signal for each of the individual haplotypes $\{(H_i)_n\}$ for $i = \{1, 2, 3, \ldots, P\}$, and n denotes the spatial position in the signal for the haplotype H_i, as mentioned above.

For a collection of reads $\mathbf{R} = \{R_1, R_2, \ldots, R_N\}$ sampled from the viral population $\mathbf{H}$, each read can be represented by a spatial signal $\{(R_i)_n\}$. The signal is non-zero for a fixed number of spatial positions equal to the read length, and is defined as described above.

1.1.3. Translation Operator for Spatial Signals

A translation operator T shifts a genomic signal H by one spatial position to the right. Mathematically, the translated signal $\{(T \cdot H)_n\}$ is defined as $(T \cdot H)[n] = H[n-1]$ for its nth sample.

A genomic signal translated by r bases to the right is denoted as $\{(T^r \cdot H)_n\}$, where $(T^r \cdot H)[n] = H[n-r]$.

The translation operator is useful as an identical subsequence of bases (or a k-mer) at multiple locations in the genome can be represented as translations of a single spatial signal.

1.1.4. Spatial Signal for Sampled Reads

A read sampled from a specific location in the genome can be represented by translation of its spatial signal by an appropriate number of bases. Thus, a single spatial signal $\{\mathbf{R}_n\}$ can be constructed for all the reads where the signal at the nth position represents the distribution of bases observed at that position in all the haplotypes. Here $\mathbf{R}[n] = (r_A[n], r_C[n], r_G[n], r_T[n])'$ where $r_A[n]$ denotes the number of aligned reads which have the base A at position n, and so on.

1.1.5. Inner Products of Spatial Signals

As the genomes are represented as 4-dimensional spatial signals, the standard definitions of vector addition and scalar multiplication apply to them. For two genomic signals X and Y defined above, the inner product of the two signals is defined as : $\langle X, Y \rangle = \sum_n \sum_{b \in \{A,C,G,T\}} x_b[n] \cdot y_b[n]$ The inner

product of two genomic signals measures the similarity of the two sequences represented by X and Y. When X, Y represent genomic signals of a single haplotype, the inner product reduces to a Kronecker delta product between the elements of X and Y at each spatial location n.

If X is a spatial signal from the sequenced reads and Y represents a haplotype H_i, then the inner product provides a measure of the concordance between the sequenced reads and the haplotype H_i.

1.2. Frames for Genomic Signals

1.2.1. Representation of k-mers of a Given Size as a Set of Signals

A set $\mathbf{C_k}$ of signals is defined as a collection of 4^k discrete 4-dimensional spatial signals. The signals in the set $\mathbf{C_k}$ have a one-to-one correspondence to the set of all possible 4^k k-mers.

Signals in the set $\mathbf{C_k}$ are denoted as $C_{k,l}$, where the index k denotes that the signal belongs to the set $\mathbf{C_k}$ and the index $l \in \{1, 2, \ldots, 4^k\}$ denotes one of the 4^k k-mers. Each signal $C_{k,l}$ is non-zero only at spatial positions $[0, (k-1)]$ and has exactly one non-zero entry in the four axes at a given spatial position.

For example, the set $\mathbf{C_1}$ consists of four spatial signals:

$$\mathbf{C_1} \equiv (\{(C_{1,A})_n\}, \{(C_{1,C})_n\}, \{(C_{1,G})_n\}, \{(C_{1,T})_n\}) \tag{2}$$

, where $\{(C_{1,A})_n\}$ is defined as follows:

$$C_{1,A}[n] = \begin{cases} (1,0,0,0) & \text{if } n = 0 \\ (0,0,0,0) & \text{otherwise} \end{cases} \tag{3}$$

The signals $\{(C_{1,C})_n\}$, $\{(C_{1,G})_n\}$, and $\{(C_{1,T})_n\}$ are defined in similar fashions where, at $n = 0$, the second, third, and fourth dimension of the 4-dimensional vector is respectively one. Thus, the signals $\{(C_{1,A})_n\}$, $\{(C_{1,C})_n\}$, $\{(C_{1,G})_n\}$, and $\{(C_{1,T})_n\}$ correspond to the 1-mers (A,C,G,T) being observed at the first position of the genome.

For $k = 2$, the set $\mathbf{C_2} \equiv (\{(C_{2,AA})_n\}, \{(C_{2,AC})_n\}, \ldots,$
$\{(C_{2,TT})_n\})$ consists of 16 spatial signals, each of which is non-zero for only two spatial positions. For example,

$$C_{2,AT}[n] = \begin{cases} (1,0,0,0) & \text{if } n = 0 \\ (0,0,0,1) & \text{if } n = 1 \\ (0,0,0,0) & \text{otherwise} \end{cases} \tag{4}$$

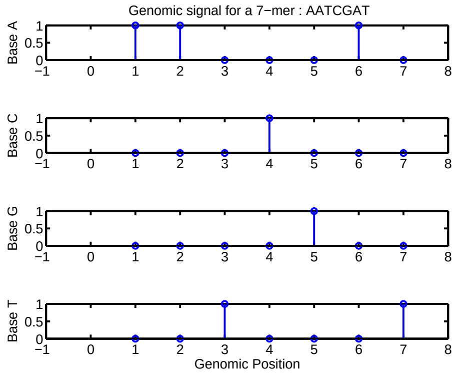

Figure 1: **Example genomic signal for a 7-mer.** An illustrative example of a 7-mer, AATCGAT, represented as a four-dimensional spatial signal. The four axis correspond to four bases in genomes (A,G,C,T). The signal is non-zero at seven spatial positions corresponding to the seven bases in the 7-mer.

and so on for the remaining 15 signals in $\mathbf{C_2}$. The signals in the set $\mathbf{C_2}$ thus correspond to observing all possible 2-mers at the first two positions in the genome.

A pictorial representation for a signal in the set $\mathbf{C_7}$ corresponding to 7-mer AATCGAT shows that the 7-mer can be trivially reconstructed from the signal by replacing the non-zero base value on the y-axis at the spatial position depicted by the x-axis (Figure 1).

1.2.2. Representation for $\mathbf{C_k}$ *from* $\mathbf{C_{k-1}}$

We can also obtain the set $\mathbf{C_k}$ of signals iteratively using the signals in the set $\mathbf{C_{k-1}}$ and the translations of the signals in the set $\mathbf{C_1}$.

$$\mathbf{C_k} \equiv \{C_{(k-1),l} + (T^{(k-1)} \cdot C_{1,b}) : \quad l \in \{A,C,G,T\}^{(k-1)}, \quad b \in (A,C,G,T)\} \quad (5)$$

where $C_{(k-1),l}$ is a signal in the set $\mathbf{C_{k-1}}$ and $(T^{(k-1)} \cdot C_{1,b})$ is the translation of the signal $C_{1,b}$ to the position $(k-1)$. In other words, the signal for k-bases in the genome can be obtained by taking a signal for the first $(k-1)$-bases and appending it with the translated version of a signal in the set $\mathbf{C_1}$. The set of signals in $\mathbf{C_k}$ thus denote the 4^k possible k-mers that exist in $\{A,C,G,T\}^k$.

1.2.3. Family of Signals from set $\mathbf{C_k}$

A family of signals is obtained by translating the signals in the set $\mathbf{C_k}$ to any spatial position r. Mathematically, $\mathbf{C_{k,\mathbb{N}}} \equiv \{T^r \cdot C_{k,l} : r \in \mathbb{N}, l \in \{A, C, G, T\}^k\}$ which corresponds to all translations of the signals in the set $\mathbf{C_k}$ by $r \in \mathbb{N}$ spatial positions. The family is indexed by three parameters: k indicating the set $\mathbf{C_k}$ of signals used, l indicating a particular signal of the possible 4^k signals in the set $\mathbf{C_k}$, and r indicating a spatial position to which the signal $C_{k,l}$ is translated.

The family of signals $\mathbf{C_{k,\mathbb{N}}}$ physically corresponds to signals for k-mers observed at any position in a genome. For example, the signal $T^r \cdot C_{k,l}$ corresponds to the signal $C_{k,l}$ translated by r bases to the right, so that it is non-zero between positions r to $r + k - 1$. In other words, it corresponds to the k-mer represented by $C_{k,l}$ starting at position r in the genome. Thus, as $C_{k,l}$ varies over the set $\mathbf{C_k}$, the family $\mathbf{C_{k,\mathbb{N}}}$ can represent all k-mers at all genomic positions. This is important as the genome of any haplotype can be represented in terms of a family of signals for a given size k.

1.2.4. Family of Generating Signals $\mathbf{C_{k,\mathbb{N}}}$ *as a Frame for Genomic Signals*

We define a function $\tilde{f}_k : H \to \mathbb{N}$, given a genomic signal H and a family $\mathbf{C_{k,\mathbb{N}}}$, as:

$\tilde{f}_k(l, r) \equiv \langle H, (T^r \cdot C_{k,l}) \rangle$, $\tilde{f}_k(l, r)$ denotes the inner-product of the signal H with the generating signal $C_{k,l}$ that has been translated by r positions, a member of the family $\mathbf{C_{k,\mathbb{N}}}$. The function $\tilde{f}_k(l, r)$ is indexed by l and r, where l ranges between 1 to 4^k and r ranges over the spatial positions.

For example, consider the projections of H onto the family $\mathbf{C_{1,\mathbb{N}}}$. Here, $\tilde{f}_1(l, r)$ denotes the unique projections of H onto the signals in the family $\mathbf{C_{1,\mathbb{N}}}$. As a signal $T^r \cdot C_{1,l} \in \mathbf{C_{1,\mathbb{N}}}$, is non-zero only at one spatial position, namely r, and the genomic signal H has only non-zero entry at each spatial position, the function $\tilde{f}_1(l, r)$ is equal to one only in the direction of the base observed in H at a position r. This allows us to uniquely express H using its projections, namely: $H[n] = \sum_{b \in \{A,C,G,T\}} \tilde{f}_1(l, r) \cdot (T^r \cdot C_{1,b})[n]$.

This is easy to see, as the members of the family $\mathbf{C_{1,\mathbb{N}}}$ are orthonormal and are non-zero at only one spatial position and for only one of the four dimensions. Each of the signal samples a single base at a single spatial position in the signal H and the collection of projections $\tilde{f}_1(l, r)$ is another way to specify the genomic signal H. This is very similar to the concept of sifting property of discrete delta functions in signal processing.

The family $\mathbf{C_{k,\mathbb{N}}}$ forms a frame for representing genomic signals Kaiser

(2010) and can be used for representing any genomic signal H. In other words, genomic signal H can be described using its projections onto the family of functions $\mathbf{C}_{\mathbf{k},\mathbb{N}}$. These projections can be used to completely specify the signal H.

In order to show that $\mathbf{C}_{\mathbf{k},\mathbb{N}}$ forms a frame, we show that Equation 1 holds for all genomic signal H with the inner-product as defined above. We show two properties for the function $\tilde{f}_k(l,r)$ that demonstrate $\mathbf{C}_{\mathbf{k},\mathbb{N}}$ is indeed a frame for genomic signals and the fact that only analyzing the k-mers present in a genome is sufficient for its representation.

Proposition 1.1. *The maximum value of the projection* $\tilde{f}_k(l,r)$ *for signal* H *from a single genome,* $\max_l \tilde{f}_k(l,r) = k$.

Proof. At position n, the signal $H[n] = (x_A[n], x_C[n], x_G[n], x_T[n])'$ from a single genome satisfies two conditions: (i) $x_b[n] \in \{0,1\}$, and (ii) $\sum_b x_b[n] = 1\ \forall\ b \in \{A,C,G,T\}$ and $n \in [0, |H|-1]$.
Similarly, for the signal $T^r \cdot C_{k,l}[n] = (y_A[n], y_C[n], y_G[n], y_T[n])$ in the frame $\mathbf{C}_{\mathbf{k},\mathbb{N}}$, two conditions are true:

$$y_b[n] = \begin{cases} \{0,1\} & \text{if } n \in [r, r+k-1] \\ 0 & \text{otherwise} \end{cases}$$

, and $\sum_b y_b[n] = 1$, $\forall\ b \in \{A,C,G,T\}$ and $n \in [r, r+k-1]$.

Using the definition of inner-products, and above four conditions, we obtain

$$\tilde{f}_k(l,r) = \sum_{n=r}^{r+k-1} \sum_b x_b[n] \cdot y_b[n] \leq k.$$

as the product $x_b[n] \cdot y_b[n]$ is either zero or one depending on whether $x_b[n]$ matches with $y_b[n]$ or not.

□

As the projection $\tilde{f}_k(l,r)$ is bounded by k for a single genome and there are only finite number of non-zero projections ($4^k \cdot |H|$ total projections) on the family $\mathbf{C}_{\mathbf{k},\mathbb{N}}$, the upper bound β for the frame definition (Equation 1) can be obtained trivially ($\beta = k$). Even for the sampled reads signal $\mathbf{R}_n$, its projections are again bounded by $k \cdot \max |\mathbf{R}[n]|$ in a $k-$spatial domain window. The lower bound α can be obtained as all non-zero signals have

projection of at least one. Thus, the bounds in the frame definition are still valid and the family of signals $\mathbf{C}_{\mathbf{k},\mathbb{N}}$ is indeed a frame.

We next show that the projection of a genomic signal H is maximized when the generating signal is for the k-mer matching the genomic signal H.

Proposition 1.2. *The k-mer u observed at position r in the signal H corresponds to the signal in the family $\mathbf{C}_{\mathbf{k},\mathbb{N}}$ for which the inner product $\langle H, (T^r \cdot C_{k,l})\rangle$ is maximized $u = \operatorname{argmax}_l \tilde{f}_k(l,r)$.*

Proof. From Proposition 1.1, it follows that the maximum value of $\tilde{f}_k(l,r)$ is achieved when both $x_b[n]$ and $y_b[n]$ from positions r to $r+k-1$ are simultaneously 1. This occurs for the signal $T^r \cdot C_{k,l}$ corresponding to the k-mer observed in H from positions r to $r+k-1$. All other signals in the family are either zero in this range or have projections less than k. □

Proposition 1.2 suggests that of all the projections of H onto the family $\mathbf{C}_{\mathbf{k},\mathbb{N}}$ in a $k-$spatial positions window, the maximum projection is on the generating signal corresponding to the k-mer of H in the same window. We refer to this projection as the *maximal projection* for a signal H in a $k-$base window. This is helpful as it gives the mathematical proof from the signals domain about something that is well known in practice, namely, that the a genome can be expressed in terms in terms of k-mers of a given length.

As the family $\mathbf{C}_{\mathbf{k},\mathbb{N}}$ forms a frame for the discrete four-dimensional spatial signals, the projections of the reads signal onto this family forms can be used to represent the signal.

1.2.5. Representation of Sampled Reads Signal $\{\mathbf{R_n}\}$ as Projections on Frames

The maximal projections of the sampled reads signal $\{\mathbf{R}_n\}$ onto the frame $\mathbf{C}_{\mathbf{k},\mathbb{N}}$ can be computed using the above definitions and we show below that they are directly proportional to the count of the k-mers present in the sampled reads. Consider the projection of the reads signal $\{\mathbf{R}_n\}$ onto a signal $T^r \cdot C_{k,l} \in \mathbf{C}_{\mathbf{k},\mathbb{N}}$: $\langle \mathbf{R}[n], (T^r \cdot C_{k,l})\rangle = \sum_i \langle R_i[n], (T^r \cdot C_{k,l})\rangle$ where the summation on the right is over all the reads. As the signal $(T^r \cdot C_{k,l})$ is non-zero only for spatial locations r to $(r+k-1)$, only reads sampled from that segment in the viral genomes would contribute terms to the summation. If we only focus on the maximal projections of the reads signal, the individual inner-products for a read $R_i[n]$ will attain their maximum value k (using propositions 1.1 and 1.2) when the generating signal $C_{k,l}$ matches the k-mer present in $R_i[n]$ in this window. Thus, for a particular generating signal $T^r \cdot C_{k,l}$, the number

of times it achieves its maximum will be exactly equal to the number of times a k-mer is observed in the all the reads at the positions r to $r + k - 1$.

If one considers all the maximal projections of the reads signal $\{\mathbf{R}_n\}$ onto the frame $\mathbf{C}_{\mathbf{k},\mathbb{N}}$, the values of the projections are in fact equal to the counts of k-mers times the constant k. This is crucial as the properties of projections of signals can now be applied to the counts of k-mers for distinguishing between erroneous and rare variant k-mers, both of which have low k-mer counts in the sampled reads.

The above equation implies that as long as the size of k is large enough that a k-mer can only be sampled from a single location in the genome, all its observed counts would contribute to exactly one generating signal $T^r \cdot C_{k,l}$ in the family $\mathbf{C}_{\mathbf{k},\mathbb{N}}$. In viral populations, where repeats are small, it is possible to choose reasonable values of k for the above to be true. Thus, the choice of k for the frame $\mathbf{C}_{\mathbf{k},\mathbb{N}}$ is important and should be large enough such that a k-mer only occurs once in the haplotypes. On the other hand, it should be smaller than the read lengths so that k-mer counting is still meaningful.

The minimum k can be approximated by ensuring that the probability of picking a string of length $|H|$ where all k-mers in it occur only once Kelley et al. (2010). Thus the probability of picking approximately $|H|$ unique k-mers out of a set of $4^{k/2}$ (considering reverse complements) should be low. We set $2 \cdot |H|/4^k \approx \epsilon$, where ϵ is a small number, to determine the smallest possible choice of k (k_{min}) for the frame $\mathbf{C}_{\mathbf{k_{min}},\mathbb{N}}$.

1.2.6. Representation of k-mers as a Series of Frames for Error Detection

The reads signal can be projected onto multiple families of signals as additional redundancy helps in reducing the noise in the signal, assuming the noise is uniformly distributed across the reads Mallat (1999). If the parameter k for a frame $\mathbf{C}_{\mathbf{k},\mathbb{N}}$ is greater than k_{min}, the maximal projections of the reads signal will be proportional to the counts of the k-mers, and one can choose multiple such frames for representing the reads. For example, given the frames $\mathbf{C}_{\mathbf{k},\mathbb{N}}$, $\mathbf{C}_{\mathbf{k'},\mathbb{N}}$, $\mathbf{C}_{\mathbf{k''},\mathbb{N}}$ for $k > k' > k'' > k_{min}$, the maximal projections of the reads signal on these three frames will correspond to the counts of the k-mers, k'-mers, k''-mers of all the reads.

The projections of the reads signals onto a series of frames can be used for detection of erroneous windows and of rare variants similar to noise removal in signal processing Unser (1995); Ron and Shen (1995). As the projections of the reads are obtained in a window of fixed size we also perform detection of rare variants and errors based on the k-mers and their projections onto a

series of frames.

For a k-mer u occurring $c(u)$ times in the reads we denote its spatial signal as $\{u_n\}$. The maximal projection of $\{u_n\}$ onto the frame $\mathbf{C}_{\mathbf{k},\mathbb{N}}$ is $k \cdot c(u)$. For k-values (k', k'' in the range $[k_{min}, k]$, the signal $\{u_n\}$ can also be projected onto the frames $\mathbf{C}_{\mathbf{k'},\mathbb{N}}$ and $\mathbf{C}_{\mathbf{k''},\mathbb{N}}$. As before, the maximal projections of $\{u_n\}$ onto these frames are equal to the counts of k'-mers and k''-mers present within u in their respective dimensions and frames.

2. References

Daubechies, I., Grossmann, A., Meyer, Y., 1986. Painless nonorthogonal expansions. Journal of Mathematical Physics 27 (5), 1271–1283.

Daubechies, I., Han, B., Ron, A., Shen, Z., 2003. Framelets: Mra-based constructions of wavelet frames. Applied and computational harmonic analysis 14 (1), 1–46.

Duffin, R. J., Schaeffer, A. C., 1952. A class of nonharmonic fourier series. Transactions of the American Mathematical Society, 341–366.

Ferreira, P., 1999. Mathematics for multimedia signal processing ii: Discrete finite frames and signal reconstruction. Nato ASI Series of Computer and Systems Sciences 174, 35–54.

Kaiser, G., 2010. A friendly guide to wavelets. Springer Science & Business Media.

Kelley, D. R., Schatz, M. C., Salzberg, S. L., et al., 2010. Quake: quality-aware detection and correction of sequencing errors. Genome Biol 11 (11), R116.

Mallat, S., 1999. A wavelet tour of signal processing. Academic press.

Ron, A., Shen, Z., 1995. Frames and stable bases for shift-invariant subspaces of $l^2(r^d)$. Canadian Journal of Mathematics 47 (5), 1051–1094.

Unser, M., 1995. Texture classification and segmentation using wavelet frames. Image Processing, IEEE Transactions on 4 (11), 1549–1560.
